# Recent advances in surgical strategies and liver transplantation for hepatoblastoma

**DOI:** 10.1002/cam4.5300

**Published:** 2022-11-16

**Authors:** Masaki Honda, Koushi Uchida, Tomoaki Irie, Kazuya Hirukawa, Masashi Kadohisa, Keita Shimata, Kaori Isono, Naoki Shimojima, Yasuhiko Sugawara, Taizo Hibi

**Affiliations:** ^1^ Department of Pediatric Surgery and Transplantation Kumamoto University Graduate School of Medical Sciences Kumamoto Japan; ^2^ Department of Surgery Tokyo Metropolitan Children's Medical Center Tokyo Japan

**Keywords:** associating liver partition and portal vein ligation for staged hepatectomy, hepatoblastoma, indocyanine green navigation surgery, liver transplantation, minimally invasive surgery

## Abstract

Hepatoblastoma (HB) is the most common malignant liver tumor in children. Although the development of treatment strategies with advances in chemotherapy has greatly improved the prognosis of HB, surgical resection and liver transplantation still play a vital role in the treatment of HB. In recent years, technological innovations have led to the development of new surgical approaches for HB. In this review, we describe the latest research on the surgical management of HB, including new imaging technologies, minimally invasive approaches, and the application of associating liver partition portal vein ligation for staged hepatectomy. We also discuss the current role of liver transplantation, use of ante‐situm or ex‐situ liver resection with auto‐transplantation, and management of metastatic HB.

## Graphical abstract

Hepatoblastoma is the most common malignant liver tumor in children. In this review, we describe the latest technologies and techniques for the surgical management of hepatoblastoma, including new imaging technologies, minimally invasive approaches, and the application of associating liver partition portal vein ligation for staged hepatectomy. We also discuss the current role of liver transplantation, liver transplant‐related surgical techniques, and management of metastatic hepatoblastoma.

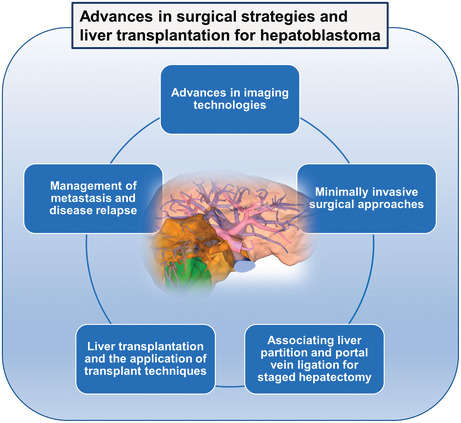

## 1 INTRODUCTION

Hepatoblastoma (HB) is the most common primary liver malignancy in children, usually occurring in the first 3 years of life.^1^ Epidemiological analyses have shown that the incidence of HB has been increasing, partially because of the improved survival of premature infants.^2^, ^3^ Over the past 3 decades, overall survival has improved from 30% to 80% following innovative advances in chemotherapy and surgical techniques, including liver transplantation (LT).^1^, ^4^, ^5^, ^6^ Disease stage and current treatment protocols depend on the degree of local tumor burden defined by the pre‐ and post‐treatment extent of disease (PRETEXT or POST‐TEXT) system.^7^ The PRETEXT group (I, II, III, or IV) is determined by the number of contiguous tumor‐free liver sections. For example, PRETXT III tumors involve either two or three liver sections with only contiguous tumor‐free section, and PRETEXT IV tumors involve all four sections. According to this system, multiple studies have shown a correlation between the PRETEXT stage and overall survival for HB patients.^4^, ^8^, ^9^ It is worth noting that advances in chemotherapy with cisplatin‐based regimens have made 50%–85% of initially unresectable HBs resectable.^10^, ^11^ However, the curability of chemotherapy‐resistant or metastatic HB is highly dependent on the achievement of radical surgical resection, including LT.^12^, ^13^ In fact, it has been reported that aggressive metastatic resection provides a good prognosis even if lung metastases, the most common form of metastasis of HB, remain after chemotherapy.

In recent years, steady progress in not only chemotherapy but also surgical methods and tools for HB treatment has been made. In this review, we focus on the latest technologies and techniques for the surgical management of HB, outlining their advantages and limitations. We also describe the current role of LT and LT‐related surgical techniques and the management of HB metastasis.

## 2 ADVANCES IN IMAGING TECHNOLOGIES

**FIGURE 1:**
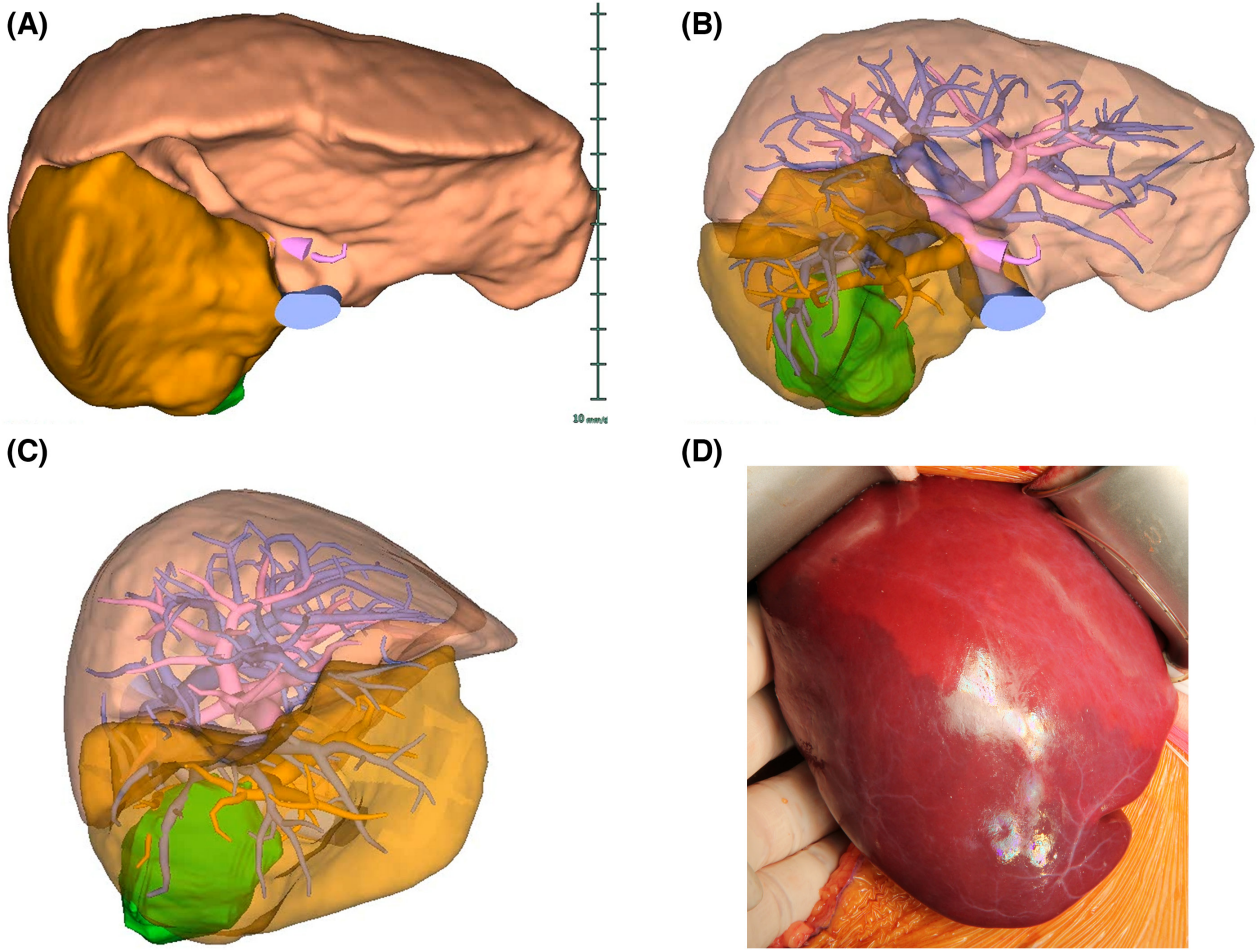
Virtual 3D imaging of liver structures. Representative (A) whole‐liver 3D imaging, (B) translucent 3D imaging, (C) right side view, and (D) actual intraoperative demarcation line. Hepatoblastoma is visualized in green, indicating its positional relationship with the portal veins (red) and hepatic veins (blue). The orange area indicates the right posterior segment

Detailed preoperative imaging is critical for performing effective surgery in HB patients. In recent years, technological advances have led to the development of 3D image‐processing software and virtual simulation of hepatectomy. These powerful tools enable 3D reconstruction of the liver and provide individual information regarding the positional relationship between the tumor and vascular structures, such as the portal vein and hepatic veins (Figure 1). In addition, 3D printing technologies have assisted in surgical planning for HB and helped improve the understanding of hepatic anatomy and tumor characteristics.^14^ Of note, the information is also useful for patient/parent education.^15^ Furthermore, it has been reported that diffusion weighted magnetic resonance imaging (MRI) is useful as a preoperative evaluation for detecting satellite lesions of HB.^16^ Meyers et al. advocated that gadoxetate disodium (Gd‐EOB‐DTPA), which is a hepatobiliary MRI contrast agent, is useful in the evaluation of HB in defining the relationship of the tumor to hepatic and portal veins, and in examining intrahepatic lesions distant from the main tumor.^17^ Gd‐EOB‐DTPA imaging also helps to differentiate focal nodular hyperplasia from the recurrent HB.

**FIGURE 2:**
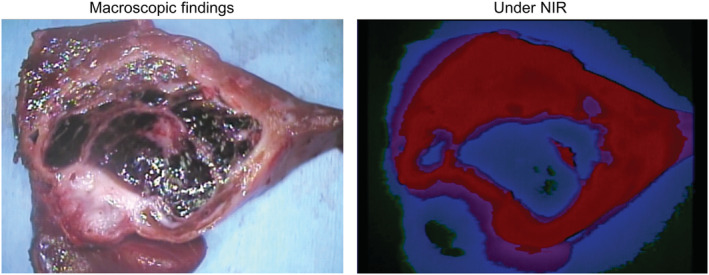
Fluorescence of indocyanine green under near‐infrared (NIR) identifies hepatoblastoma in the excised liver specimen

In addition to innovations in preoperative imaging technology, accurate intraoperative evaluation of tumor localization is extremely important to achieve complete surgical resection for HB. Souzaki et al. demonstrated the usefulness of an intraoperative augmented reality navigation system for detecting tumor‐related anatomy during pediatric surgery, particularly for endoscopic surgery.^18^ Moreover, fluorescence‐induced indocyanine green (ICG) navigation surgery has recently been applied to identify the localization of hepatic tumors intraoperatively. ICG emits light with a wavelength of approximately 840 nm when illuminated with near‐infrared light.^19^ During intraoperative cholangiography, Ishizawa et al. discovered that the liver tumor was fluorescing under near‐infrared light.^20^ They identified that ICG was retained longer in hepatocellular carcinoma tissues than in the hepatic parenchyma. Subsequently, the fluorescent imaging method for liver tumors using ICG was applied to identify the liver metastasis of colorectal or pancreatic cancer and intrahepatic cholangiocarcinoma.^21^ Kitagawa et al. used an ICG navigation system during thoracotomy for HB metastasis and identified 250 fluorescence‐positive lesions in 10 patients with 37 operations.^22^ Surprisingly, the diameter of the smallest detectable lesion was 0.062 mm, and the positive predictive value was 88.4%. On the other hand, one of the potential problems was false positives, and indeed 29 of the 250 lesions (11.6%) proved not to be HB pathologically. Application of the ICG navigation system in HB patients has been reported to be useful for detecting tumors and confirming complete resection during surgery^22^, ^23^, ^24^, ^25^, ^26^ (Figure 2). This system is also useful for detecting satellite HB lesions.^27^ However, it is worth noting that the ICG navigation system is insufficient for detecting tumors located at a depth of more than 10 mm from the organ surface.^24^ On the basis of reports to date, the recommended interval between ICG injection (0.5 mg/kg) and HB resection is approximately 72 h for the liver or peritoneum and around 24 h for the lungs.^28^ Currently, the ICG navigation system should not replace preoperative diagnostic imaging and intraoperative inspection and palpation, but it complements these techniques to achieve the complete resection of HB.

## 3 MINIMALLY INVASIVE SURGICAL APPROACHES

Laparoscopic approaches for liver surgery are indicated primarily for lesions located in the anterolateral segments of the liver (S2‐6), and wedge resections, partial hepatectomies, and left lateral segmentectomies have been widely performed in adult patients.^29^ Intermittent inflow occlusion and the use of low central venous pressure anesthesia are very effective for the proper control of bleeding during laparoscopic hepatectomy. Laparoscopic resection of HB was first described in 2000 by Waldhausen et al.,^30^ and the usefulness and safety of this technique have since been reported.^31^, ^32^ As a device of the procedure, radiofrequency‐assisted pre‐coagulation can be useful for homeostasis and oncological safety during the resection of large HBs.^32^ Recently, Kwon et al. reported their experiences with laparoscopic liver resection in children, including 13 HB patients.^33^ No mortality was observed in their cohort, and it was proposed that (1) selecting patients by a thorough review of preoperative images, (2) using non‐laparoscopic ultra‐sonography to determine the location of target vessels during laparoscopic liver resection, (3) setting the Cavitron ultrasonic surgical aspirator amplitude and aspiration level lower than those in laparotomy, (4) ligating the Glisson structures distal from the confluence, and (5) dissecting the target vessel at least 7 mm from the ligating point were the critical points to performing laparoscopic liver resection safely with minimal bleeding.

Recently, robotic surgery has been applied in various abdominal surgeries.^34^ Among them, the Da Vinci surgical system, which has a 3D HD magnification imaging system and an artificial wrist arm with a flutter filter function, is the most popular and enables detailed and accurate surgery.^35^ It has been reported that robotic‐assisted liver resection has the advantages of reducing postoperative complications, including bleeding and biliary fistula, without significant differences in the amount of intraoperative blood loss compared with laparoscopic liver surgery.^36^ In reports on HB, robot‐assisted S5 hepatectomy with gallbladder preservation in a 3‐year‐old child^37^ and robotic right hepatectomy for an adult HB patient^38^ have been described. The application of robotic surgery in HB is still in the preliminary stage, but it is an area in which future development is desired.

## 4 ASSOCIATING LIVER PARTITION AND PORTAL VEIN LIGATION FOR STAGED HEPATECTOMY (ALLPS) FOR HB

An insufficient future liver remnant (FLR) can lead to post‐hepatectomy liver failure. To achieve the complete and safe resection of extensive liver tumors, the ALLPS procedure has been performed to increase the volume of FLR for adult patients in small‐for‐size FLR settings. The original ALLPS procedure is a two‐stage hepatectomy that combines first‐stage portal vein ligation and in situ splitting of the liver, followed by liver removal 1–2 weeks later.^39^ To reduce the disadvantages and morbidity of ALLPS, modified ALPPS procedures have been performed as alternatives, including hepatic parenchymal transection in the first stage and portal vein embolization the next day or the transection of 50%–80% of the liver parenchyma.^40^, ^41^ In 2014, Chan et al. reported the first case of an HB patient who underwent the ALPPS procedure.^42^ FLR volumetry showed a significant increase in FLR/estimated total liver volume from 21.2% to 30.2% on the seventh post 1st operative days. After that, a right trisectionectomy was performed safely, and the postoperative course was uneventful. Pediatric case series consisting of three HB patients who underwent the ALPPS procedure also showed a favorable rapid increase in FLR.^43^ To date, challenging but successful cases, such as modified ALPPS in a small infant (54 days old) with HB,^44^ pure laparoscopic 1st stage partial ALPPS,^45^ and monosegment 6 ALPPS for a POST‐TEXT IV HB,^46^ have been reported. As a modified procedure, two‐stage laparoscopic hepatectomy for giant HB with selective hepatic artery ligation and liver partial partition has also been introduced.^47^ However, there are reports of rapid tumor recurrence in residual livers after ALPPS in adult and pediatric patients.^48^, ^49^ Although it is difficult to assess whether the ALPPS procedure played a vital role in recurrence, a potential contribution of the immune response that promotes liver regeneration to rapid tumor growth cannot be ruled out. These findings indicate that the ALPPS procedure can sometimes be a double‐edged sword. ALPPS may be indicated in less than 10% of HB cases^50^; however, because none of the previous publications have described in detail the reasons for unresectability, it remains speculative. Because pediatric patients are known to tolerate major hepatic resection, and as little as 20%–25% of FLR is sufficient, ALPPS should only be indicated for selected patients who can potentially achieve curative resection without the need for LT.

## 5 CURRENT STATUS OF LT AND THE APPLICATION OF TRANSPLANT TECHNIQUES TO EXTEND CONVENTIONAL SURGICAL MARGINS

LT is an important option for unresectable HB. In the case of central POST‐TEXT III and IV HB, primary LT is recommended because the tumor may remain histologically present even if the absence of imaging findings. Additionally, advanced HB patients with poor chemosensitivity should be prioritized for primary LT.^51^ With the progress in perioperative care and the refinement of surgical skills, previous studies have shown an improvement in the 5‐year overall survival rate from 50% to 90% in HB patients who underwent LT.^5^, ^52^ The National Cancer Database of the United States also showed favorable outcomes for HB patients who received LT regardless of vascular invasion.^13^ Furthermore, Kasahara et al. advocated the use of living donor LT (LDLT) as a valuable alternative method for unresectable HB patients because it enables the optimal timing of LT without a delay between the completion of chemotherapy and the planned LT.^53^ A nationwide survey from Japan showed that the independent risk factors for HB recurrence after LDLT were a high alpha‐fetoprotein level at diagnosis (500,000 ng/ml), the presence of extrahepatic lesions before LDLT, and a high alpha‐fetoprotein level at LDLT (4000 ng/ml).^54^ In recent years, the application of pediatric living donor domino LT, improvement in perioperative management for ABO‐incompatible LDLT, and development of split deceased donor LT have increased the opportunity to obtain grafts for LT, especially in areas where there is a shortage of organs for transplantation.^55^, ^56^, ^57^ The latest multicenter study using the Society of Pediatric Liver Transplantation (SPLIT) database revealed that tumor extent or the presence of metastasis and vascular involvement did not affect event‐free survival for HB transplants, suggesting that transplant outcomes can be favorable despite a high tumor burden.^58^ Moreover, the risks of infection and renal injury were higher in malignant compared with non‐malignant pediatric LT.^58^ Determining the appropriate immunosuppressive regimen with renal function protection and antitumor effects is a topic for future studies. Uchida et al. reported that early inflow exclusion using a temporary portocaval shunt during LT might improve recurrence‐free survival by decreasing blood loss and tumor dissemination with the preservation of renal function.^59^ When major vessels were invaded by the tumor, vein graft replacement or portal venous tumor thrombectomy was applied successfully.^60^, ^61^, ^62^ Even more surprising is that multi‐visceral transplantation achieved complete gross resection of extensive HB with multiple organ and vascular involvement, providing long‐term disease‐free survival (4.5 and 7.8 years) in pediatric patients with no other surgical options.^63^ On the basis of a multi‐center retrospective analysis, Pondrom et al. indicated that tumor rupture might be predictive of poor prognosis with a risk of peritoneal relapse, but it should not be a contraindication for LT.^64^ In 2004, an era in which chemotherapy regimens and surgical skills for HB were developing, Otte et al. reported an overall survival rate of only 30% for 41 HB patients who underwent a salvage LT.^5^ However, the latest Japanese multi‐center study showed that there was no significant difference in the 1‐ and 5‐year survival rates between HB patients undergoing primary LT and those who received salvage LT for tumor recurrence (89.7%, 81.6% vs. 88.0%, 76%; *p* = 0.526).^65^ These data are similar to the latest SPLIT results showing 85% and 74% salvage transplant event‐free survival rates at 1 and 3‐years, respectively.^58^ Although an issue of salvage LT still remains debatable, these findings support the feasibility of salvage LT in selected patients with localized HB recurrence.

LT should be avoided if surgical resection is expected to have similar therapeutic effects in terms of the need for lifelong immunosuppressive treatment, risk of LDLT donors, and medical cost. Several groups have reported favorable outcomes for advanced HB patients treated with complex surgical resections under the appropriate conditions.^66^, ^67^, ^68^, ^69^ In some cases, local recurrence can be controlled by combining resection with chemotherapy even if the microscopic resection margins are positive after extreme hepatectomy for advanced stage HB.^66^, ^70^ It is worth noting that preoperative trans‐catheter arterial chemoembolization and/or trans‐arterial radioembolization with yttrium‐90 might help render tumors accessible to resection in unresectable HB.^71^, ^72^ Surgical skills related to transplant surgery are applied in selected cases to extend conventional margins of hepatic resection.^73^ Table 1 summarizes the recent publications on the application of transplant techniques in HB. Angelico et al. reported a case of ante‐situm liver resection (transection of the suprahepatic inferior vena cava [IVC] and anterior rotation of the liver) and IVC replacement under hypothermic cardiopulmonary bypass for a huge right lobe HB with tumoral thrombi extending into the IVC and right atrium.^77^ The IVC was replaced with an aortic graft from a blood‐group compatible deceased donor. Around the same time, a successful case of ex vivo liver resection and auto‐transplantation with cardiopulmonary bypass was reported for a similarly complicated case.^78^ At the end of hepatic resection, portal vein and vena cava catheterization and ex vivo hepatectomy to remove part of the external tumor thrombus were performed. Prior to auto‐transplantation, tumor thrombus in the IVC and right atrium was also completely removed through cardiopulmonary bypass at low cardiac arrest. These techniques are feasible options for pediatric patients with HB that is considered unresectable by conventional surgical intervention.^79^ However, it is also necessary to consider that anticoagulant therapy, such as systemic heparinization, is required to perform the procedure, which may impair hemostasis. One of the key considerations in deciding whether to choose extended hepatectomy or LT is the difference in long‐term outcomes/complications between the two techniques. Late complications of extreme hepatectomy include biliary complications and vascular complications (portal hypertension or hepatic congestion). Busweiler et al. reported that out of 73 cases of hepatectomy for HB, 9 (12.3%) developed biliary complications and 2 (2.7%) developed vascular complications.^74^ Two patients with vascular complications finally underwent LT (7 days and 9 years after hepatectomy). On the other hands, late complications of LT include biliary complications, vascular complications, chronic rejection, and side effects of immunosuppression therapy such as renal dysfunction, diabetes, hypertension, and posttransplant malignancies.^75^, ^76^

**TABLE 1:** Application of transplant techniques to extend conventional surgical margins in hepatoblastoma

Case	Age (years)	PRE‐TEXT	POST‐TEXT	Procedure	Type of resection	Vascular reconstruction	Follow‐up month	Outcome	Reference
1	0	III	III	Right hepatectomy	Ante situm	IVC reconstruction with deceased donor aortic graft	8	Alive	Angelico, et al.^74^
2	3	NA	NA	Right‐extended hepatectomy, IVC resection	Ante situm	IVC reconstruction with deceased donor vein graft	24	Alive	Schlegel, et al.^75^
3	8	NA	NA	Left‐extended hepatectomy, IVC resection	Ante situm	IVC reconstruction with deceased donor vein graft	6	Alive	Schlegel, et al.^75^
4	1	IV	III	Resection of segment IV, V, VIII, and removal of tumor thrombosis in IVC	Ex situ, auto‐transplantation	HV, IVC, and PV reconstruction	1	Alive	Shi, et al.^76^

Abbreviations: HV, hepatic vein; IVC, inferior vena cava; NA, not available; PV, portal vein.

Currently, we are witnessing a paradigm shift caused by the concept of transplant oncology. The surgical approach (super‐extended resections with the application of transplant techniques, such as ante‐situm resection and ex‐vivo resection + auto‐transplantation, vs. LT) should be determined ideally by the oncological eliminability of the tumor. The recent advances in chemotherapeutic regimens,^80^ molecular/genetic profiling,^81^ and cancer immune microenvironment^82^ are expected to pave the way to ultimately achieving a cancer‐specific survival rate of 100%, regardless of resection or LT. Because LT is an important treatment option for HB patients whose resectability is unclear, it is desirable to perform HB treatment in close contact with facilities that have sufficient experience with both hepatectomy and LT in pediatric patients.^83^

## 6 MANAGEMENT OF METASTASIS AND DISEASE RELAPSE

Approximately 20% of patients with HB have metastatic lesions, especially lung metastases, at diagnosis.^84^ For HB with synchronous metastasis, neoadjuvant cisplatin‐based chemotherapy has enabled the complete remission of lung metastasis in more than 50% of patients.^85^, ^86^, ^87^ Despite the refinement of protocols for HB treatment, children with metastatic HB suffer from a lower event‐free survival rate.^88^, ^89^ Active resection for residual lung metastatic lesions is recommended in cases of chemotherapy failure.^86^, ^90^ In a valuable case report, Horiike et al. showed that aggressive treatment with surgical resection and chemotherapy are also effective options for ruptured HB with disseminated tumors and lung metastasis in infants.^91^ As described, ICG navigation surgery for metastasectomy would be a feasible option for detecting the nodules of lung metastases accurately. Moreover, McDaniel et al. reported a technique for identifying lung metastases during thoracoscopic surgery using a CT‐guided localization method combining a methylene blue blood patch and hook wire.^92^ Combining ICG navigation and CT‐guided marking provides a powerful approach.^93^ Meanwhile, some surgeons consider the combination of methylene blue blood patch and ICG to be contraindicated due to concerns that the injected dye may obscure the ICG fluorescence, and this issue would require further investigation.

The International Childhood Liver Tumor Strategy Group (SIOPEL) reported that disease relapse in HB occurs in less than 12% of patients after complete remission.^94^ In addition, most relapses occur in the residual liver and lungs. Li et al. reported that age ≥3 years, PRETEXT IV, and metastatic disease are independent risk factors of HB recurrence after complete remission.^95^ Despite disease relapse, JPLT and SIOPEL studies showed that over half of the relapsed patients could achieve a second complete remission with chemotherapy and surgical resection.^94^, ^96^ Moreover, long‐term survival was achieved in 8 of 10 patients with lung relapse by pulmonary metastasectomy.^97^ Therefore, repeat multiple thoracotomies are recommended until complete resection is achieved to prevent HB recurrence and prolong disease‐free survival. ICG techniques can also be used for the adequate guidance of a second resection for relapsed HB. It has also been reported that re‐transplantation could be performed in selected patients with multiple intragraft HB metastases and peritoneal recurrence,^25^ demonstrating the importance of aiming for complete tumor resection under any circumstances.

## 7 CONCLUSIONS AND FUTURE PERSPECTIVES

In recent years, in addition to the refinement of chemotherapy protocols for HB patients, advances in surgical strategies in the field of hepato‐biliary and transplant surgery have improved the outcomes of HB treatment. The use of perioperative imaging technology, minimally invasive surgical approaches, and ALLPS are new fields in HB treatment that have attracted attention, and further development is expected in the future. Furthermore, it is expected that better treatment results can be achieved for HB patients by carefully combining LT with meticulous perioperative management, applying surgical techniques related to transplant surgery, and appropriately treating metastases and recurrent lesions.

## AUTHOR CONTRIBUTIONS

**Masaki Honda:** Conceptualization (lead); data curation (lead); project administration (lead); resources (lead); supervision (lead); validation (lead); writing – original draft (lead); writing – review and editing (lead). **Koushi Uchida:** Data curation (supporting); validation (supporting); writing – review and editing (supporting). **Tomoaki Irie:** Data curation (supporting); validation (supporting); writing – review and editing (supporting). **Kazuya Hirukawa:** Data curation (supporting); validation (supporting); writing – review and editing (supporting). **Masashi Kadohisa:** Data curation (supporting); validation (supporting); writing – review and editing (supporting). **Keita Shimata:** Data curation (supporting); validation (supporting); writing – review and editing (supporting). **Kaori Isono:** Conceptualization (supporting); data curation (supporting); validation (supporting); writing – review and editing (supporting). **Naoki Shimojima:** Data curation (supporting); resources (supporting); validation (supporting); writing – review and editing (supporting). **Yasuhiko Sugawara:** Data curation (supporting); validation (supporting); writing – review and editing (supporting). **Hibi Taizo:** Data curation (supporting); resources (supporting); supervision (supporting); validation (supporting); writing – original draft (supporting); writing – review and editing (supporting).

## FUNDING INFORMATION

None.

## CONFLICT OF INTEREST

All the authors declare that they have no conflicts of interest.

## ETHICS STATEMENT

Ethical approval was not needed for this review.

## Data Availability

The data that support the findings of this study are available from the corresponding author upon reasonable request.
